# SARS-CoV-2 Induced Herpes Virus Reactivations and Related Implications in Oncohematology: When Lymphocytopenia Sets in and Immunosurveillance Drops Out

**DOI:** 10.3390/microorganisms11092223

**Published:** 2023-09-01

**Authors:** Luca Roncati, Elizabeth Sweidan, Cyrielle Tchawa, Greta Gianotti, Gianluca Di Massa, Flavia Siciliano, Ambra Paolini

**Affiliations:** 1 Institute of Pathology, Department of Laboratory Medicine and Anatomical Pathology, University Hospital of Modena—Polyclinic, 41124 Modena, Italy; 2 Department of Surgery, Medicine, Dentistry and Morphological Sciences with Interest in Transplantation, Oncology and Regenerative Medicine, University of Modena and Reggio Emilia, 41121 Modena, Italy; 3 Graduate School of Anatomical Pathology, Department of Medicine and Surgery, University of Parma, 43121 Parma, Italy; 4 Graduate School of Medical Oncology, Department of Maternal, Infant and Adult Medical and Surgical Sciences, University of Modena and Reggio Emilia, 41121 Modena, Italy; 5 Unit of Diagnostic Hematology, Department of Laboratory Medicine and Anatomical Pathology, University Hospital of Modena—Polyclinic, 41124 Modena, Italy

The severe acute respiratory syndrome, coronavirus 2 (SARS-CoV-2), is a positive-sense single-stranded ribonucleic acid (RNA) virus contagious in humans and responsible for the ongoing coronavirus disease 2019 (COVID-19) [1]. First identified in Wuhan, China, the World Health Organization declared the outbreak a pandemic on 11 March 2020 [2]. To date, this disease has caused more than 6.9 million deaths [3].

SARS-CoV-2 mainly spreads via close contact and aerosols or respiratory droplets produced when speaking, breathing, exhaling, coughing, or sneezing [4]. The virus enters human cells via the interaction between its spike protein and angiotensin-converting enzyme 2 (ACE2) receptors, ubiquitous throughout the body [5].

In 67–90% of the patients affected by severe COVID-19, lymphocytopenia occurs, a well-known marker of impaired cellular immunity; both killer T cells and helper T cells have been found to decrease in these circumstances [6]. In addition, white pulp and lymphoid tissue depletion have been reported in the literature [7]. Among the pathogenetic mechanisms to explain lymphopenia and lymphodepletion, there is a direct cytotoxic action of SARS-CoV-2 related to the ACE2-dependent or ACE2-independent entry into lymphocytes [6].

With the loss of immunosurveillance, latent pathogens in the body can be reactivated, as is the example of herpes viruses. They are a family of deoxyribonucleic acid (DNA) viruses, of which nine are known to primarily infect humans, and five cause extremely common diseases, such as orolabial and genital herpes due to human herpes virus 1 (HHV1) and human herpes virus 2 (HHV2), chickenpox and shingles from human herpes virus 3 (HHV3), and mononucleosis and mononucleosis-like syndrome from human herpes virus 4 (HHV4) and human herpes virus 5 (HHV5) [8]. Over 90% of adults have been infected with at least one of these strains; depending on the virus, latent cells include neurons, monocytes, and B and T lymphocytes (Table 1).

Much of the literature discloses infective herpetic reactivations in the course of COVID-19 [9,10,11,12,13,14,15,16,17,18,19,20,21,22]; surprisingly, they have also been reported after COVID-19 vaccination, based on nucleoside-modified messenger RNAs (modRNAs) and adenoviral vectors [23,24,25,26,27,28,29,30,31,32,33,34,35,36,37,38,39,40,41,42,43,44,45,46,47,48,49]. In addition, HHV5, alias Epstein–Barr virus (EBV), and HHV8, alias Kaposi’s sarcoma-associated herpes virus, are two notorious oncoviruses. The former is responsible for EBV-positive Burkitt’s lymphoma, EBV-positive Hodgkin lymphoma, EBV-positive diffuse large B cell lymphoma (DLBCL), extranodal NK/T cell lymphoma nasal type, EBV-associated aggressive NK cell leukemia, angioimmunoblastic T cell lymphoma, post-transplant lymphoproliferative disorder, and nasopharyngeal carcinoma, while the latter for Kaposi’s sarcoma, primary effusion lymphoma, and multicentric Castleman’s disease (Table 1).

In very rare circumstances of immunodeficiency, e.g., the acquired immune deficiency syndrome (AIDS), they may act synergistically as in the case of EBV-positive HHV8-associated large B cell lymphoma with plasmablastic differentiation [50], recently encountered during our diagnostic practice on the bone marrow biopsy from a 50-year-old female lymphopenic COVID-19 patient, pancreas and kidney transplant recipient for about 20 years due to type 1 diabetes mellitus, with a rapidly lethal course (Figure 1).

Similarly, we had previously diagnosed on autoptic specimens an EBV-positive DLBCL involving the whole organism, even the lungs, in a 78-year-old male lymphopenic patient with SARS-CoV-2 infection (Figure 1). Moreover, we had reported the fatal case of a 70-year-old male patient co-affected by severe COVID-19 and EBV-positive Hodgkin lymphoma [51]. Other authors have described these associations [52,53,54,55,56,57,58,59,60,61,62,63,64,65,66,67] following COVID-19 vaccination with modRNA and adenoviral vector-based vaccines [68,69,70,71], which appear worthy of further larger-scale surveys.

The hypothesis that other DNA oncoviruses, such as human papillomavirus (HPV), may also take advantage of the immune system exhaustion induced by COVID-19 is under investigation [72], as known HPV can reactivate in the course of AIDS or graft-versus-host disease [73,74,75]. From preliminary data in a lymphopenic setting, COVID-19 can lead to rapid progression of HPV-positive cervical intraepithelial neoplasia toward microinvasive carcinoma [76]. Therefore, this further aspect should be deeply explored in the context of cervical cancer screening programs.

## Figures and Tables

**Figure 1:**
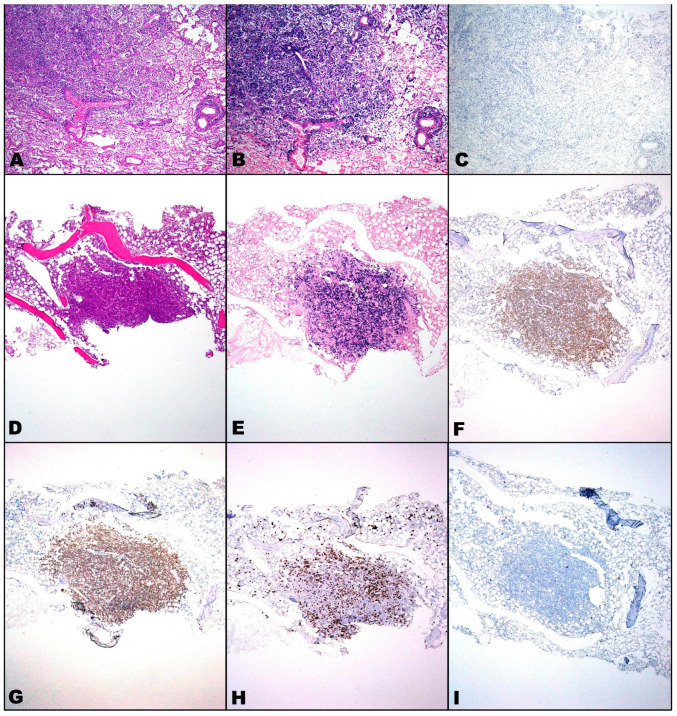
EBV-positive DLBCL lymphoma disseminated to the lungs [(**A**), hematoxylin and eosin, 40×], resulted intensely blue-stained with EBV-encoded RNA (EBER) probe [(**B**), in situ hybridization (ISH), 40×] and completely negative for HHV8 immunohistochemistry (IHC) [(**C**), 13B10 clone, 40×; chromogen: 3,3′-diaminobenzidine (DAB)], in a 78-year-old male COVID-19 patient; on the death day blood tests revealed lymphopenia (320 µL) of both killer and helper T cells. Bone marrow biopsy from a 50-year-old female COVID-19 patient, transplant bearer, showing EBV-positive HHV8-associated large B cell lymphoma with plasmablastic differentiation [(**D**), hematoxylin and eosin, 40×; (**E**), positive blue-stained EBER ISH, 40×; (**F**), positive brown-stained anti-HHV8 DAB IHC, 13B10 clone, 40×; (**G**), positive brown-stained anti-MUM1 DAB IHC, EP190 clone, 40×; (**H**), positive brown-stained anti-CD138 Syndecan-1 DAB IHC, B-A38 clone, 40×; (**I**), negative anti-CD20 DAB IHC, L26 clone, 40×]; on the death day blood tests revealed lymphopenia (330 µL) and tacrolimus concentration in normal range (6.01 ηg/mL).

**Table 1:** Names, acronyms, synonyms, main diseases, latency cells, and transmission routes of the nine viruses belonging to the Herpesviridae family that infect humans.

Name, Acronym & Synonym	Diseases	Latency	Transmission
HHV1 alias HSV1 (Herpes Simplex Virus 1)	Oral herpes	Neurons	Close contact
	Genital herpes	(sensory)	(oral and sexual)
	Herpes keratitis	(ganglia)	
HHV2 alias HSV2 (Herpes Simplex Virus 2)	Oral herpes	Neurons	Close contact
	Genital herpes	(sensory)	(oral and sexual)
	Herpes keratitis	(ganglia)	
	Mollaret’s meningitis		
HHV3 alias VZV (Varicella Zoster Virus)	Chickenpox	Neurons	Respiratory and
	Shingles	(sensory)	close contact
		(ganglia)	(oral and sexual)
HHV4 alias EBV (Epstein–Barr Virus)	Infectious mononucleosis (IM)	B cells	Close contact,
	Lymphoproliferative disorders		transfusions,
	Inflammatory pseudotumor		tissue transplant
	Nasopharyngeal carcinoma		and congenital
HHV5 alias CMV (Cytomegalovirus)	IM-like syndrome	Monocytes	Saliva, urine,
	Retinitis		blood and milk
HHV6 (A & B) (Human Betaherpesvirus 6A & 6B)	Sixth disease (roseola infantum	T cells	Respiratory and
	or exanthem subitum)		close contact
HHV7 (Human Betaherpesvirus 7)	IM-like syndrome	T cells	Respiratory and
	Hepatitis		close contact
HHV8 alias KSHV (Kaposi’s Sarcoma Associated Herpesvirus)	Kaposi’s sarcoma	B cells	Close contact
	Primary effusion lymphoma		(sexual) and
	Multicentric Castleman’s disease		saliva (?)
